# Antioxidant, anti-acetylcholinesterase, and anti-amyloid-β peptide aggregations of hispolon and its analogs in vitro and improved learning and memory functions in scopolamine-induced ICR mice

**DOI:** 10.1186/s40529-024-00443-x

**Published:** 2024-12-18

**Authors:** Chang-Hang Yang, Cai-Wei Li, Yi-Yan Sie, Liang-Chieh Chen, Yu-Hsiang Yuan, Wen-Chi Hou

**Affiliations:** 1 Graduate Institute of Pharmacognosy, Taipei Medical University, Taipei, 110 Taiwan; 2 Clinical Drug Development of Herbal Medicine, College of Pharmacy, Taipei Medical University, Taipei, 110 Taiwan; 3 School of Medicine, College of Medicine, National Sun Yat-Sen University, Kaohsiung, 804 Taiwan; 4 Traditional Herbal Medicine Research Center, Taipei Medical University Hospital, Taipei, 110 Taiwan

**Keywords:** Acetylcholinesterase, amyloid-β_1−42_-peptide aggregations, Hispolon, Scopolamine

## Abstract

**Background:**

Hispolon, one of bioactive phenolic compounds from a medicinal mushroom of sang-huang (*Phellinus linteus*) has been reported to exhibit anticancer and anti-inflammatory activities. The Alzheimer’s disease (AD) is ranked one of the top ten leading causes of death worldwide. Little is known about the effects of hispolon on delaying AD progression.

**Results:**

The hispolon (No.1) and its six structural analogs (No.2 to No.7) were assayed by antioxidant, anti-acetylcholinesterase activities and anti-amyloid-β_1-42_-peptide aggregations. The No.1, No.6, and No.7 were selected for further molecular docking with acetylcholinesterase and core fragments of amyloid-β-peptide, and also showed capacities to recover cell viabilities in methylglyoxal-treated SH-SY5Y cells and also to enhance neurite outgrowths in PC12 cells. The daily pre-treatments of No.1, No.6, and No.7 for 10-days (40 mg/kg/day) showed to improve learning dysfunctions in scopolamine-induced ICR mice by passive avoidance tests.

**Conclusion:**

The hispolon in the fungus sang-huang might be beneficial to develop functional foods or as lead compounds for treating degenerative disorders.

**Supplementary Information:**

The online version contains supplementary material available at 10.1186/s40529-024-00443-x.

## Background

The World Health Organization reported at 2023 that over 55 million people have suffered dementia globally in a nearly 10-million case increase every year and the number of dementia people is expected to 82 million by 2030 and 152 million by 2050, among those who suffered Alzheimer’s disease (AD) accounts for 60–70% of total cases (World Health Organization 2003). The AD and other dementias is ranked in the 7th position of the top ten leading causes of death worldwide in 2021, and also ranked in the 4th position of the leading causes in the high-income countries nearly four-fold increases of 2000 (World Health Organization 2024), and no effective medicine is available on delaying AD progression. It was reported that the neuron damages resulted in brain changes of the AD patients get started 20 years or more before the memory loss, and the damaged numbers and the damaged areas in the brain affect the progression in the AD symptoms (Serrano-Pozo et al. 2011; Tarawneh and Holtzman 2012; Alzheimer’s Association Report 2023; National Institute on Aging). Except the genetic factor of the APOE4 homozygotes (Fortea et al. 2024), it was calculated that the AD prevalence in aged persons were 47%, 75%, and 80% of dementia cases in ages between 70 and 79, between 80 and 89, and over 90, which the old age is the main risk factor (Plassman et al. 2007). Therefore, AD makes global impacts on building up social assistance systems, financial burden supports, elder cares, and medical developments.

The AD patients are characterized by the memory loss with common pathologies of synaptic failures and neuron deaths associated with extracellular amyloid β plaques and neurofibrillary tangles (Querfurth and LaFerla 2010; Craig et al. 2011; Selkoe and Hardy 2016), however, no successful drug is available in delaying progressions of cognitive dysfunctions in persons with mild cognitive impairments to early AD patients. The clinical drugs for AD patients include acetylcholinesterase (AChE) inhibitors, such as the FDA-approved donepezil, rivastigmine, galantamine developed by cholinergic hypothesis (Alzheimer’s & Association 2024); and the FDA-approved memantine, a *N*-methyl-D-aspartate receptor antagonist by blocking excitotoxic effects of glutamate (Witt et al. 2004). These available drugs are only symptomatic treatments and have shown short-term cognitive improvements, but have no long-term effects in delaying cognitive declines. The mainstream theory of AD etiology over the past 30 years is the amyloid cascade hypothesis, and the small molecules targeting processing enzymes associated with amyloid β peptide generations or macromolecules of humanized anti-amyloid monoclonal antibodies most were failed to improve cognition function (McGleenon et al. 1999; Čolović et al. 2013; Perneczky et al. 2023; Cummings et al. 2024). Two recent anti-amyloid monoclonal antibodies have received more attentions for passing the main endpoint in delaying cognitive declines in clinical trials. One is the US FDA-accelerated approval of aducanumab (Aduhelm^Ⓡ^, Biogen Inc., MA) on June 2021, which is designed specifically to amino acid residues of 3 to 7 in the Aβ peptide and targeted to aggregated Aβ (Arndt et al. 2018); and the other is US FDA approval of lecanemab (Leqembi^Ⓡ^, Eisai Co., Tokyo and Biogen Inc., MA) on July 2023, which is designed and targeted to soluble Aβ protofibrils (van Dyck et al. 2023), and the lecanemab intravenously every 2 weeks (10 mg/kg of body weight) in the 18-month phase-III trial have showed to 27% less cognitive decline in a clinical dementia rating-sum of the boxes (CDR-SB) change between baseline and last visit compared to those in the placebo. It is the first successful signs of AD drug developments and efforts should be continued. It is recognized that a single hypothesis cannot fully explain the pathogenesis of AD and can involve protein abnormalities of Aβ peptide aggregates and Tau protein phosphorylation, the loss of cholinergic neurons and synaptic failure, metal ion accumulations, oxidative stress, neuro-inflammation, and mitochondrial dysfunction (Jack et al. 2009; Weinreb et al. 2016; Monteiro et al. 2023; Osborne et al. 2023). The major reactive dicarbonyl species in the human body of methylglyoxal, an intermediate of reducing sugar metabolism, interacted with amine groups in *N*-terminal or side-chain AA residues of proteins to form irreversible advanced glycation end-products, which could change conformations, functions, and degradations of proteins or enzymes (Kikuchi et al. 2003). It was also reported that the reactive methylglyoxal enhanced to crosslink Aβ aggregates or accelerate Aβ fibril accumulations, which the increased ROS productions and elevated oxidative stress might result in neuronal cell deaths and cognitive impairments (Kikuchi et al. 2003; Gella and Durany 2009; Allaman et al. 2015). Therefore, the integrated therapies shall be developed as new routes for AD treatments by combining approved drugs of specific targets based on amyloid cascade hypothesis and/or cholinergic hypothesis with multifaceted targets of biological activities of natural compounds. Several natural compounds have reported to exhibit multifaceted targets associated with AD pathologies. The demethylcurcumin, a naturally existed curcumin analog, has showed dose-dependent radical scavenging activities (Liu et al. 2019), AChE inhibitory activities, anti-Aβ peptide_1 − 42_ aggregations, and neuroprotective activities against 6-hydroxydopamine-induced cell deaths in vitro, and has improved learning and memory functions in scopolamine-induced impaired-memory ICR mice (Liu et al. 2020). The piceatannol (a resveratrol analog) and its dimer of scirpusin B have showed dose-dependent radical scavenging activities, AChE inhibitory activities, anti-Aβ_1 − 42_ peptide aggregations, neuroprotective activities against Aβ_25 − 35_ peptide-induced cell deaths in vitro, and also have improved learning and memory functions in scopolamine-induced impaired-memory ICR mice (Sie et al. 2023a, b). The tellimagrandin II (an ellagitannin) isolated from water caltrop hulls or vitisin A (a resveratrol tetramer) isolated from roots or stems of herbal plants of *Vitis thunbergii* var. *taiwaniana* have showed dose-dependent AChE inhibitory activities and anti-Aβ peptide_1 − 42_ aggregations, and also have improved functions evaluated by passive avoidance test or Morris water maze in scopolamine-induced impaired-memory ICR mice (Chen et al. 2022).

The fungus sang-huang (*Phellinus linteus*), a kind of medicinal mushroom grown in the tree trunk, has been classified as top grade in the Ancient China Pharmacopoeia of Shen Nong Ben Cao Jing for benefits to the five internal organs, detoxification, and promoting Qi, and now is reported to exhibit biological activities of antitumor, antioxidant, immunomodulation, hepatoprotection, blood glucose regulations, and anti-inflammation (Hsieh et al. 2013; Chen et al. 2016), such as the polysaccharide fractions of hot-water extraction of sang-huang showed DPPH radical scavenging activities, anti-lipid peroxidation, and ferrous ion cheating activities (Kozarski et al. 2011); the extracellular polysaccharide from the submerged culture of *Phellinus linteus* showed hypoglycemic activities in streptozotocin-induced models (Kim et al. 2001). Hispolon is one of active phenolic compounds isolated from sang-huang (Sarfraz et al. 2020), which has been identified to exhibit biological activities such as anti-cancer cells based on changes of cell viabilities and anti-inflammatory activities via TNF-α-induced NF-κB activation in vitro (Ravindran et al. 2010), hepatoprotective activities against CCl_4_-induced liver injuries of rats via increased levels of antioxidant enzymes (superoxide dismutase, catalase, and glutathione peroxidase) and reductions of inflammation index and lipid peroxidations (Huang et al. 2012), and anti-analgesic effects in acetic acid-induced writhing response and λ-carrageenan-induced paw edema of mice (Chang et al. 2011). The hispolon and its two structural analogs (dehydroxyhispolon and hispolon monomethyl ether) showed anti-inflammatory and anti-apoptotic activities by inhibiting nitric oxide productions and iNOS protein expressions and elevating heme oxygenase-1 protein expressions in lipopolysaccharide-treated BV-2 microglia cells (Wu et al. 2017). Therefore, in this study, hispolon (No.1) and its six structural analogs (No.2 to No.7), including one additional hydroxy group at phenyl ring of C-2 or C-5 or different amounts methoxy groups at phenyl ring of hispolon, were used to determine multifaceted targets associated with AD pathologies, including DPPH radical scavenging activity, oxygen radical absorbance capacity (ORAC), anti-nitric oxide productions, AChE inhibitory activities, and anti-Aβ_1 − 42_-peptide aggregations. Hispolon (No.1), 2-hydroxy hispolon (No.6), and 2-hydroxy hispolon monomethyl ether (No.7) were selected for molecular docking with AChE and Aβ_1 − 42_ peptide, neuroprotection against methylglyoxal-treated SH-SY5Y neuron cell models, and in vivo oral administrations in scopolamine-induced ICR mice to evaluate learning behavior improvement by passive avoidance tests. These results might suggest that hispolon in the fungus sang-huang might have neuroprotective activities and delaying cognitive impairments, which be beneficial to develop functional foods or as lead compounds for degenerative disorders.

## Methods

### Materials

**Fig. 1:**
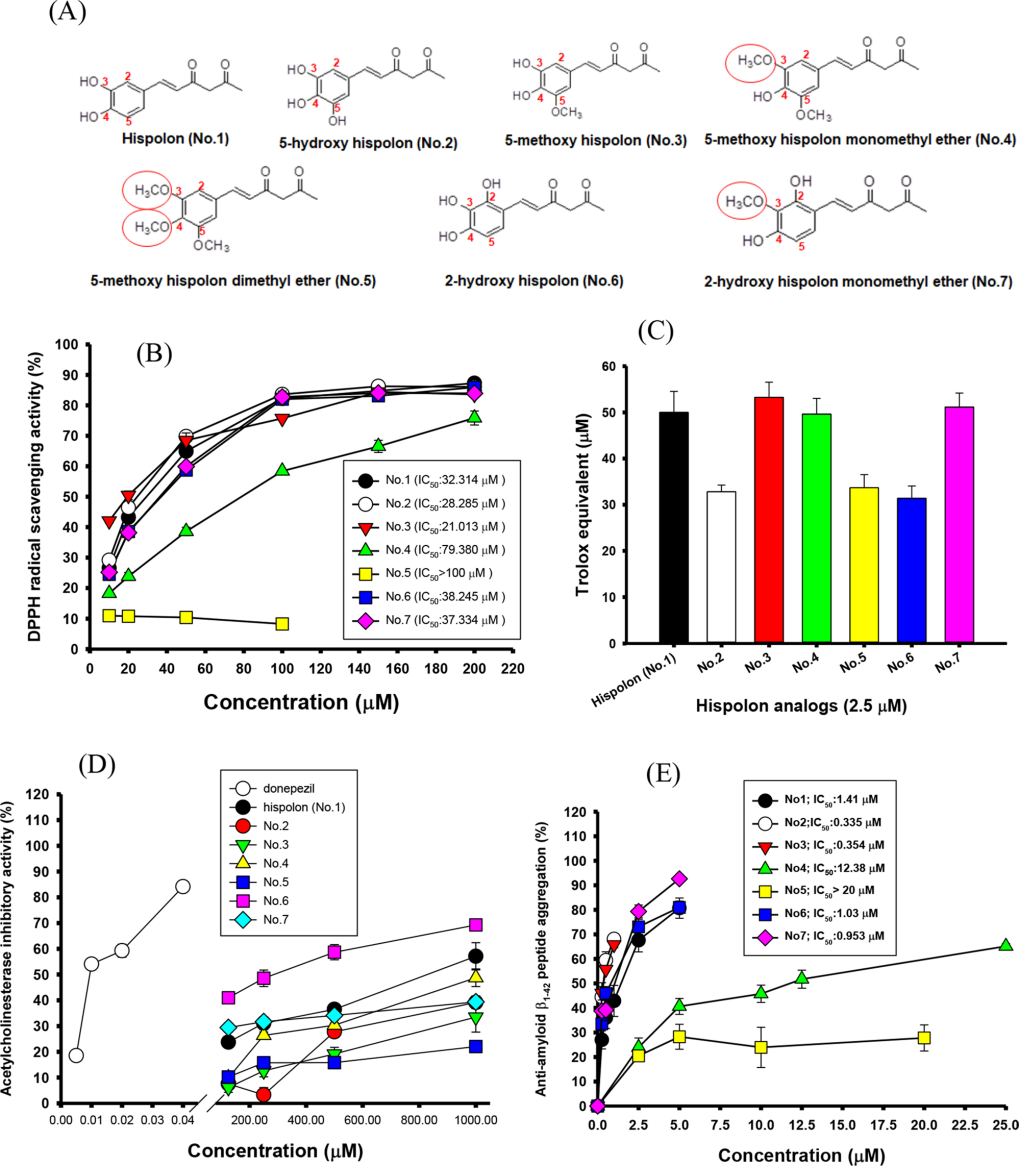
The structures of hispolon (No.1) and its six structural analogs used in the present study (**A**). Effects of hispolon (No.1) and its six structural analogs on (**B**) DPPH scavenging activities; (**C**) oxygen radical absorbance capacity (ORAC) and expressed as Trolox equivalents (µM); (**D**) acetylcholinesterase inhibitory activities; and (**E**) anti-amyloid-β_1−42_ peptide aggregations detected by thioflavin T fluorescence. The samples used in the present study included hispolon (No.1), 5-hydroxy hispolon (No.2), 5-methoxyhispolon (No.3), 5-methoxyhispolon monomethyl ether (No.4), 5-methoxyhispolon dimethyl ether (No.5), 2-hydroxy hispolon (No.6), and 2-hydroxy hispolon monomethyl ether (No.7). The donepezil was used as a positive control of the acetylcholinesterase inhibitor. Data were expressed as mean ± SD of three independent experiments, and calculated the concentration for half-inhibition (IC_50_)

Hispolon (No.1), 5-hydroxy hispolon (No.2), 5-methoxyhispolon (No.3), 5-methoxyhispolon monomethyl ether (No.4), 5-methoxyhispolon dimethyl ether (No.5), 2-hydroxy hispolon (No.6), and 2-hydroxy hispolon monomethyl ether (No.7) were purchased from Laila Impex Co. (Vijayawada, India) with purity higher than 99% (Fig. 1A). The OxiSelect™ Assay Kit (STA-345) for ORAC determination was from Cell Biolabs Inc. (San Diego, CA). The BCA ^TM^ protein assay kit and was from Thermo Fisher Scientific Inc. (Rockford, IL). The recombinant human AChE (P22303) was from R&D Systems Inc. (Minneapolis, MN). The 2,2’-azo-bis(2-amidinopropane)dihydrochloride (AAPH), Aβ_1− 42_ peptide, acetylthiocholine iodide, bovine serum albumin (BSA), 5,5’-dithiobis(2-nitrobenzoic acid) [DTNB], 3-(4,5-dimethyl-2-thiazolyl)-2,5-diphenyl-2 H-tetrazolium bromide (MTT), 2,2-diphenyl-1-picrylhydrazyl (DPPH), lipopolysaccharide (LPS), methylglyoxal, nerve growth factor (NGF, lyophilized powder, N0513), phosphate-buffered saline (PBS), poly-L-lysine solution, scopolamine hydrobromide, and thioflavin T (ThT) were purchased from Sigma Chemical Co. (St. Louis, MO). The donepezil hydrochloride was from Tokyo Chemical Industry Co. (Tokyo, Japan). The RAW 264.7 macrophages were from the Bioresource Collection and Research Center, the Food Industry Research and Development Institute (Hsinchu, Taiwan). The human SH-SY5Y neuroblastoma cells (CRL-2266) and rat PC12 pheochromocytoma cells (CRL-1721) were from American Type Culture Collection (Manassas, VA). The fetal bovine serum (FBS), Dulbecco’s modified eagle medium (DMEM), DMEM/F-12 medium, RPMI-1640 medium, and Opti-MEM medium were purchased from Gibco BRL Life Technologies (Grand Island, NY).

### DPPH radical scavenging activity

The DPPH radical scavenging activities of hispolon and its analogs were determined following a previous report (Lu et al. 2012). The 60 µL of different concentrations of samples (10, 20, 50, 100, 150, and 200 µM diluted with DMSO) were added into a 96-well plate, and then 140 µl of 100 µM DPPH in methanol was added under light protection for 20 min. The changes of absorbance at A517nm were measured, and DMSO was used instead of a sample solution in the control group. The scavenging activity of the DPPH radical (%) was calculated with the following equation: (Control-sample)/control ×100%. The concentration for half-inhibition (IC_50_) of the DPPH radical scavenging activity was calculated from each linear equation by three test concentrations (20, 50, and 100 µM) and their radical scavenging activities.

### ORAC activity

The ORAC activities of hispolon and its analogs on delaying AAPH radical-mediated fluorescent decays were determined using the OxiSelect™ assay kit by following the previous reports (Liu et al. 2019, 2020). The Trolox (5, 10, 20, 40, and 60 µM) were used to block fluorescent decays during 60-min by determining the ratio of Ex480 nm/Em520 nm. The standard plot was performed by using Trolox concentrations and area under curve of fluorescent decay (AUC) during each Trolox additions. Each sample at 2.5 µM was used to block fluorescein oxidation during 60-min and then calculated the AUC. The ORAC activity of each hispolon and its analogs at 2.5 µM concentration was interpolated the standard curve and was expressed as µM Trolox equivalents (µM TE).

### AChE inhibitory activity

The recombinant human AChE inhibitory activities of hispolon and its analogs were determined following previous reports (Liu et al. 2020; Chen et al. 2021, 2022; Sie et al. 2023a, b). The 100 µL of reaction mixture contained hispolon and its analogs (the final concentrations of 125, 250, 500, and 1000 µM in DMSO), the diluted recombinant human AChE (0.079 µg per ml in 100 mM phosphate buffer, pH 7.5), and 100 mM phosphate buffer (pH 7.5), which was placed at 4 °C for 15 min. The donepezil (5, 10, 20, and 40 nM) was used as the positive control. The AChE substrates of acetylthiocholine iodide was added for another 15 min, and the DTNB coupling reagents was added and the mixture was recorded at 405 nm for 10 min. The DMSO instead of sample solution was used as the blank. AChE inhibition (%) was calculated by [(A405_blank_-A405_sample_)/(A405_blank_)] ×100%.

### Anti-Aβ1 − 42 peptide aggregations in vitro

The anti-Aβ_1–42_ peptide aggregations of hispolon and its analogs were determined the fluorescent changes following the previous reports by the ThT binding assays (Liu et al. 2020; Chen et al. 2021, 2022; Sie et al. 2023a, b). The preparation of Aβ_1–42_ peptide stock solution was following the previous report (Liu et al. 2020). The working solution of Aβ_1 − 42_ peptide (10 µM) was mixed with 10 µM ThT solution, and hispolon and its analogs (No.1, 0.25, 0.5, 1.0, 2.5, and 5.0 µM; N0.2 and No.3, 0.25, 0.5, and 1.0 µM; No.4, 2.5, 5.0, 10, 12.5, and 25 µM; No.5, 2.5, 5.0, 10, and 20 µM; No.6 and No.7, 0.25, 0.5, 2.5, and 5.0 µM) in the 96-well was sequentially added, which the mixture was shaking continuously at 37 °C for 24 h. The DMSO was used instead of the sample solution as the control. The fluorescence ratios of Ex_440nm_/Em_486nm_ were recorded at the time of 0 h and 24 h. The inhibition against Aβ aggregation (%) was calculated as [(E_control,24−h_-E_control,0−h_)- (E_sample,24−h_-E_sample,0−h_)/(E_control,24 h_-E_control,0−h_)] ×100%. The IC_50_ of anti-Aβ_1 − 42_ peptide aggregation was calculated from each linear equation of three test concentrations (No.1, 0.5, 1.0, 2.5, and 5.0 µM; N0.2 and No.3, 0.25, 0.5, and 1.0 µM; No.4, 10, 12.5, and 25 µM; No.6 and No.7, 0.25, 0.5, and 2.5 µM).

### Anti-nitric oxide productions in lipopolysaccharide-treated RAW264.7 macrophages

The cultural protocol of RAW264.7 macrophages and LPS treatments were following the previous report (Liu et al. 2020). The 5 × 10^4^ macrophages/well were seeded in the 96-well plate and cultured for 24 h at 37 °C under the humidified atmosphere and 5% CO_2_. The cells were pre-treated with hispolon (No.1, 2.5, 5, 10, and 20 µM), No.2, No.3, No.4, and No.5 (5, 10, and 20 µM), and No.6 and No.7 (5, 10, 20, and 40 µM) for 24 h. The LPS (500 ng/ml) was then added to the pre-treated RAW264.7 macrophages for another 24 h for nitric oxide productions. For nitric oxide productions in LPS-treated RAW264.7 macrophages, the culture supernatant (100 µl) was mixed with 100 µl of Griess reagent at room temperature for 30 min. The absorbance at 550 nm was measured and expressed as relative nitric oxide productions (the control was recognized as 100%). The MTT stained method was used to measure cell viability (Wang et al. 2006). The sample pretreated-RAW264.7 cells were stained with MTT solution (500 µg/mL), which the formazan was dissolved by 200 µL DMSO, and the absorbance at 570 nm was measured, and was expressed relative to the blank (%).

### Molecular dockings

The BIOVIA software Discovery Studio (DS) was used to perform docking analyses of AChE and Aβ_1−42_ peptide with hispolon (NO.1), No.6 and No.7 in silico following the previous report (Sie et al. 2023a, b). The crystal structures (https://www.rcsb.org/structure/) of AChE (PDB ID: 4EY7) and the Aβ_1−42_ peptide (PDB ID: 1Z0Q) were acquired from Protein Data Bank (PDB), and the protein structures were prepared by the automatic ligand preparation function in DS Macromolecules Tools for protonation, removal of water molecules, and mismatched residues fixing. The AChE binding site was defined as 10 Å from the co-crystallized ligand. Validation of the docking protocol by using donepezil. The co-crystallized ligand was re-docked to confirm the docking protocol of AChE. The binding site for the Aβ_1−42_ peptide was automatically detected and established to encompass the entire protein within a sphere of a 10 Å radius. The docking compounds of hispolon and its analogs of No.6 and No.7 were prepared by the ligand preparation function in the Small Molecules Tools of DS for protonation and fixing valence. The prepared compounds were docked to the binding sites using the CDOCKER Docking Optimization function with default settings. The program generated poses for each compound and created an interaction profile using the DS interaction analysis function. The final binding poses for AChE and Aβ_1−42_ were selected based on interaction energy from all docking poses.

### Neuroprotective activities in methylglyoxal-treated SH-SY5Y neuronal cell models

The different concentrations of methylglyoxal (100, 200, 400, 500, and 600 µM) were used to treat SH-SY5Y neuronal cells, and the cell viabilities were determined. The neuroprotective activity of hispolon and its two analogs (No.6 and No.7) on 500 µM methylglyoxal-treated human SH-SY5Y neuroblastoma cells were following the previous report (Sie et al. 2023b; Chen et al. 2022). The SH-SY5Y cells (1 × 10^4^ cells/well) were seeded onto a 96-well microplate and cultured in a DMEM/F-12 medium containing 10% FBS for 24 h at 37 °C under a humidified atmosphere and 5% CO_2_. The cultural medium was removed, and the different concentrations of hispolon (No.1), No.6, and No.7 or 0.1% DMSO (the control and the blank) were added and incubated at 37 °C in a humidified atmosphere with 5% CO_2_ for 24 h. The treated medium was removed, washed with PBS, and 500 µM MGO in PBS were added to the medium for another 24 h culture. The equal aliquot of PBS was added in the blank. Then, the MTT was used to evaluate cell viability, and the absorbance at 570 nm was determined (Wang et al. 2006). Before MTT assays, the cell morphologies of methylglyoxal-treated SH-SY5Y cells were photographed using an inverted microscope (200-fold magnifications, ECLIPSE TS100, Nikon Instruments Inc., Tokyo, Japan).

### Neurite outgrowths in PC12 rat pheochromocytoma cell models

The stimulated activities of neurite outgrowths of hispolon (No.1) and its two analogs (No.6 and No.7) in PC12 rat pheochromocytoma cell lines were following the previous report (Liao et al. 2012). The PC12 cells (5 × 10^3^/well) were seeded in poly-L-lysine coated 96-well plate and cultured in RPMI-640 medium supplemented with 10% horse serum, 5% FBS, 100 U/ml penicillin and 100 mg/l streptomycin for 24 h at 37 °C under the humidified atmosphere and 5% CO_2_. For enhanced neurite outgrowths of PC12 cells, each sample (final concentrations of 1 and 2 µM in DMSO) was added into Opti-MEM medium supplemented with 0.5% FBS for a 3-day culture, and the NGF (100 ng/ml) in the same culture condition was used as the positive control (Hu et al. 2018). The DMSO was used instead of the sample solution as the blank. After an interval of 3 days, the cell morphologies were photographed using an inverted microscope (200-fold magnifications, ECLIPSE TS100, Nikon Instruments Inc., Tokyo, Japan). For calculating average neurite lengths, at least 30 neurites were selected and analyzed by the Image J 1.54 software (Liao et al. 2012).

### Improved learning behaviours in scopolamine-induced cognitive dysfunction ICR mice

The animal experiments were under the approval of the Institutional Animal Care and Use Committee of Taipei Medical University (LAC-2017-0360), and animal protocols were following the previous reports using scopolamine-induced cognitive dysfunction ICR mice (Liu et al. 2020; Chen et al. 2021, 2022; Sie et al. 2023a, b). The 5-week-old (*N* = 30) male ICR mice were purchased from the Laboratory Animal Center in College of Medicine, National Taiwan University, and were housed in wire-bottomed stainless-steel cages and had free access to normal feeds (Prolab^Ⓡ^ RMH2500, MO) and water, which these cages were placed in an environmental controlled space under a 12-h light/dark cycle. After at least one-week acclimation, the mice were randomly divided into six groups, including the blank, the control, the positive control (donepezil-intervened), and the sample pre-treated groups (No.1, No.6, and No.7 groups). In the first stage of animal experiments during day 1 to day 10, the ICR mice in each sample groups were pre-treated orally with 40 mg/kg of hispolon (No.1) and No.6 and No.7 once daily for 10 days by gavage feeding from day 1 to day 10. For mice in the blank, the control, and the positive control, mice were orally received the same volume of distilled water once daily for 10 days in the parallel experiments. In the second stage of animal experiments during day 11 to day 15, mice in the blank, the control, and the sample groups were treated as reported in the first stage, and mice in the positive control group were treated with donepezil (5 mg/kg) orally once a day by gavage feeding, and 30 min later, mice in the control, sample groups, and positive control were injected intraperitoneally with scopolamine (1 mg/kg, dissolved in PBS), and mice in the blank were injected with the same volume of PBS. At day 13 to day 15, after being injected with scopolamine or the same volume of PBS for 30 min, mice in each group were evaluated learning behaviours by the passive avoidance test. The passive avoidance apparatus, a computerized chamber (PACS-30, Columbus Instruments Inc., Columbus, OH) separated by a sliding guillotine door, is composed of a LED light box and a dark box. At the first day of the acquisition trial (day 13), the guillotine door was opened and each mouse was placed in the LED light box. In case the mouse entered the dark box, the automatic and computerized sliding guillotine door was closed, the entered mouse in the dark box received an electric foot shock (0.3 mA for 3 s) delivered via the wired metal floor and then sent back to the cage. The staying time in the light box (the step-through latency, sec) was recorded. The protocol in the second day (day 14) was the same as the first day. If the mouse stayed in the light box longer than 300 s, and the mouse was expelled into light box and received the same electric foot shocks. At the third day (day 15) of the retention trial, each mouse was placed again in the light box, and the staying time in the light box was recorded. The maximum time was 300 s.

### Statistical analyses

The present data were expressed as mean ± SD of three independent experiments, and the learning behaviours in the passive avoidance of animal experiments were also expressed as mean ± SD. The Student’s *t*-test was used to compare between [(the control) vs. (each sample treatment)] or [(the control) vs. (the blank)]. It was considered statistically a significant difference when *P* < 0.05 *, or *P* < 0.01**, or *P* < 0.001***. The one-way analysis of variance (ANOVA) and the *post hoc* Tukey’s test were used to compare multiple groups of average neurite lengths and step-through latency (sec) in scopolamine-induced ICR mice. It was considered statistically a significant difference (*P* < 0.05) when the different uppercase letters (for neurite outgrowths, the retention trial) or lowercase (for the acquisition trial) in each bar. The GraphPad Prism Window 6.0 software (San Diego, CA, USA) was used to perform statistical analyses.

## Results

### Effects of hispolon and its six analogs on antioxidant activities in vitro

Figure 1(A) showed the structures of hispolon (No.1) and its analogs (No.2 to No.7), and the methoxy group in the phenyl ring was marked by the red circle. These compounds contain different hydroxy and methoxy groups, which may have different biological activities. Figure 1(B) showed the DPPH radical scavenging activities. Except No.5, all compounds exhibited dose-dependent DPPH radical scavenging activities with different capacities. The IC_50_ of DPPH radical scavenging activities of hispolon (No.1), No.2, No.3, No.4, No.5, No.6, and No.7, respectively, were 32.31 µM, 28.29 µM, 21.01 µM, 79.38 µM, higher than 100 µM, 38.25 µM, and 37.33 µM. The DPPH scavenging capacities of test compounds were No.3 > No.2 > No.1 > No.7 ≅ No.6 > > No.4 > > No.5. The supplementary Figure S1 showed the fluorescent changes (Ex480 nm/Em520 nm) of hispolon (No.1) and its analogs (No.2 to No.7) at concentration of 2.5 µM on inhibiting AAPH-mediated fluorescent decays in ORAC activity determinations. The blank was the curve of AAPH-mediated fluorescent decays, and the positive control of Trolox at 40 µM, and hispolon (No.1) and its analogs (No.2 to No.7) showed to delay the fluorescent decays in time-dependent manners. It was found that hispolon (No.1) and its analogs (No.2 to No.7) at 2.5 µM showed radical scavenging activities and time-dependent delays of fluorescent reductions, and the calculated ORAC activities were showed in the Fig. 1(C) as the Trolox equivalents (µM). The ORAC activities of hispolon (No.1) and its analogs (No.2 to No.7) at 2.5 µM could be divided into two groups, one was about 50 µM equivalents of Trolox of No.1 (50.00 ± 4.54), No.3 (53.27 ± 3.26), No.4 (49.63 ± 3.40), and No.7 (51.16 ± 3.02), the other was about 30 µM equivalents of Trolox of No.2 (32.8 ± 1.45), No.5 (33.67 ± 2.85), and No.6 (31.39 ± 2.68). It was clear that hispolon (No.1) and its analogs (No.2 to No.7) were 14-fold or 20-fold better than that of the Trolox in the ORAC activities.

### Effects of hispolon and its six analogs on anti-nitric oxide productions, AChE inhibitory activities and anti-Aβ1 − 42 peptide aggregations in vitro

The supplementary Figure S2(A) showed the anti-LPS-induced nitric oxide productions of hispolon and its six analogs in RAW264.7 macrophages. It was clear that the LPS (500 ng/ml) enhanced significantly (*P* < 0.01) productions from 30% (the medium only as the blank) to 100% (the control).

Compared to the control, the addition of hispolon and its six analogs showed dose-dependent reductions and had significantly different in nitric oxide productions (**P < 0.01 or ***P < 0.001), especially the anti-nitric oxide productions of the hispolon (No.1). The supplementary Figure S2(B) showed effects of different concentrations of hispolon and its six analogs on cell viabilities of RAW2647.7 macrophages. Except hispolon (No.1), the other six analogs (No.2 to No.7) showed minor cytotoxicities toward RAW264.7 macrophages. The reductions of nitric oxide productions of hispolon (No.1) higher than 5 µM might be from cytotoxicities toward RAW264.7 macrophages. It was found that under no apparent toxicities (*P* > 0.05, ns; supplementary Figure S2B) of hispolon (No.1) at low concentration of 2.5 µM (supplementary Figure S2A) showed significantly reductions (*P* < 0.001) in nitric oxide productions from100–70%. Figure 1(D) showed dose-dependent AChE inhibitory activities of hispolon (No.1) and its analogs (No.2 to No.7) in vitro during 125 to 1000 µM. The clinical drug of donepezil was used as the positive control and showed dose-dependent AChE inhibitory activities with IC_50_ of 9.19 nM. It was clear that the donepezil showed powerful AChE inhibitory activities and better than those of hispolon and its analogs in the present study. Among hispolon and its analogs, the analog No.6 and hispolon (No.1) showed the first two potent AChE inhibitory activities with IC_50_ of 286.03 and 828.0 µM, respectively; and analogs of No.4, No.7, No.2, No.3, and No.5 were less AChE inhibitory activities with IC_50_ higher than 1000 µM. Figure 1(E) showed the anti-Aβ_1 − 42_ peptide aggregations of hispolon (No.1) and its analogs (No.2 to No.7) by thioflavin T methods in vitro, which three groups could be classified, the first group included No.2, No.3, No.7, No.6, and No.1 with IC_50_ around 1.0 µM, the second group included No.4 with IC_50_ of 12.4 µM, and the third group included No.5 with IC_50_ higher than 20 µM. The IC_50_ of anti-Aβ_1 − 42_ peptide aggregations of No.2, No.3, No.7, No.6, and No.1 were 0.34, 0.35, 0.95, 1.03, and 1.41 µM, respectively. Based on the results of above-mentioned Alzheimer’s disease-associated biological activities, the hispolon (No.1), No.6, and No.7 were selected for further investigations.

### Molecular docking of AChE or Aβ1 − 42 peptide with hispolon and its two analogs

**Fig. 2:**
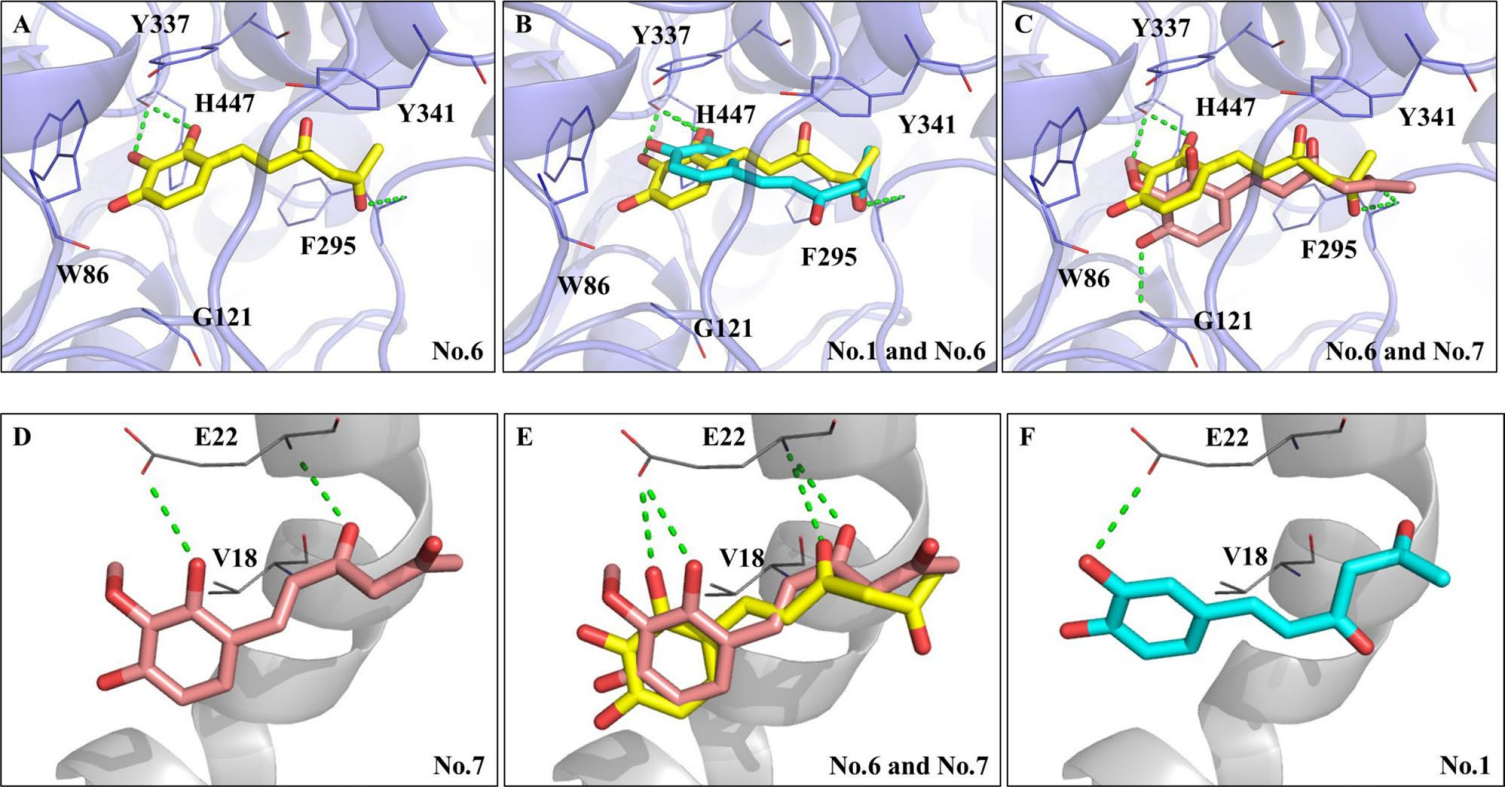
Molecular docking analyses of hispolon and its two analogs (No.6 and No.7) with acetylcholinesterase [AChE, PDB ID: 4EY7, (**A**) to (**C**)] or Aβ_1−42_ [PDB ID:1Z0Q, (**D**) to (**F**)]. (**A**) Docking poses of analog No.6 (yellow) in the active site of AChE (light blue). Superimposed docking poses of (**B**) analogs of No.6 and hispolon (No.1, cyan) (salmon), and (**C**) analogs of No.6 and No.7 (salmon) in AChE active site. (**D**) Docking poses of analog No.7 (salmon) in Aβ_1−42_ (gray). Superimposed docking poses of (**E**) analogs of No.7 and No.6 (yellow) and (**F**) hispolon (No.1, cyan) in Aβ_1−42_. Hydrogen bonds are represented as green dash lines. Residues are labeled as shown. The figures were created using Pymol

The orders of AChE inhibitory activities of hispolon (No.1) and its two analogs were No.6 > No.1 > No.7 (Fig. 1D). Therefore, these three molecules were subjected to molecular docking analysis using the computational tool Discovery Studio (DS). The supplementary Figure S3 showed the validation of the docking protocol by using donepezil as the ligand. The co-crystallized ligand was re-docked to confirm the docking protocol of AChE. It was found that these three molecules occupied the active site of AChE (light blue color) and displayed similar conformations (Fig. 2A and B, and 2C). In the complex formed by analog No. 6 (yellow color) and AChE (Fig. 2A), the C-2, C-3, and C-4 hydroxy groups of No.6 extended to the esteratic site (ES) of AChE where acetylcholine was hydrolyzed. The hydroxy groups at C-2 and C-3 positions of phenyl ring formed two hydrogen bonds with ES site of the amino acid (AA) residue H447 (green dash line, Fig. 2A). Additionally, analog No.6 also formed a hydrogen bond with the AA residue F295 in the catalytic anionic subsite. Aside from polar contacts, analog No. 6 made π-hydrophobic interactions with AA residue Y337 of AChE, which helped to stabilize the No.6/AChE complex. Figure 2B showed the superimposed dockings of analog No.6 (yellow color) and hispolon (No.1, cyan color) with the AChE active site. Hispolon (No.1), with a similar structure to analog No.6, resulted in a conformation change due to the lack of a C-2 hydroxy group in the phenyl ring. As a result, it only formed one hydrogen bond with AA residue H447, but it still formed another hydrogen bond with AA residue F295. Figure 2C showed the superimposed dockings of analog No.6 (yellow color) and analog No.7 (salmon color) with the AChE active site. Analog No.7 formed a hydrogen bond with AA residue F295 and also a hydrogen bond with AA residue G121. It was noted that the presence of C-3 methoxy group of the phenyl ring led to steric hindrance and resulted in the loss of polar contact with AA residues in the ES site and π-hydrophobic interactions with other AA residues. Supplementary Table S1 showed the interaction energy of the docking poses of hispolon (No.1) and its two analogs with AChE. It was found that the No.6/AChE showed the lowest interaction energy (-38.5581 Kcal/mol) among these three docking models and had the more stable complex, which matched the highest AChE inhibitory activities among test samples in the present study.

The orders of anti-Aβ_1 − 42_ peptide aggregations of hispolon (No.1) and its two analogs were No.7 ≅ No.6 > No.1 (Fig. 1E). Therefore, these three molecules were docked into Aβ_1−42_, and occupied the same segment of the Aβ_1−42_ helix (gray color), which is part of the aggregation core of Aβ (Fig. 2D and E, and 2F). It was reported that the AA residues 16 to 21 of the Aβ_1 − 42_ peptide were the core segments of Aβ self-aggregations (Enache et al. 2016). Analog No.7 (salmon color, Fig. 2D) not only formed two hydrogen bonds (green dash line) with AA residue E22 via the C-2 hydroxy group in the phenyl ring and the ketone groups, but also created a hydrophobic interaction with AA residue V18. The similar interactions were noted between analog No.6 (yellow color) and Aβ_1−42_ peptide (Fig. 2E), indicating that analogs No.6 and No.7 exhibited comparable activity in anti-Aβ_1−42_ peptide aggregations. Due to differences in the position of hydroxy groups in the phenyl ring, the docking position of hispolon was slightly altered (Fig. 2F). Hispolon (No.1) only generated one hydrogen bond with AA residue E22, which might result in a weaker anti-Aβ aggregations. Indeed, the C-2 hydroxy group in the phenyl ring showed more suitable for the anti-Aβ_1 − 42_ peptide aggregations. Supplementary Table S1 showed the interaction energy of the docking poses of hispolon (No.1) and its two analogs with the segments of Aβ_1−42_ peptide. It was found that the interaction energy of each complex of No.1/Aβ_1−42_, No.6/Aβ_1−42_, and No.7/Aβ_1−42_ were − 20.1893, -18.0291, and − 20.3564 Kcal/mol, respectively. The more stable complex of No.7/Aβ_1−42_ had the lowered interaction energy compared to the complex of No.6/Aβ_1−42_, which resulted in the potent breakers to inhibit Aβ aggregations.

### Effects of hispolon and its two analogs against methylglyoxal-induced SH-SY5Y cell deaths and stimulated neurite outgrowths in PC12 cell models

**Fig. 3:**
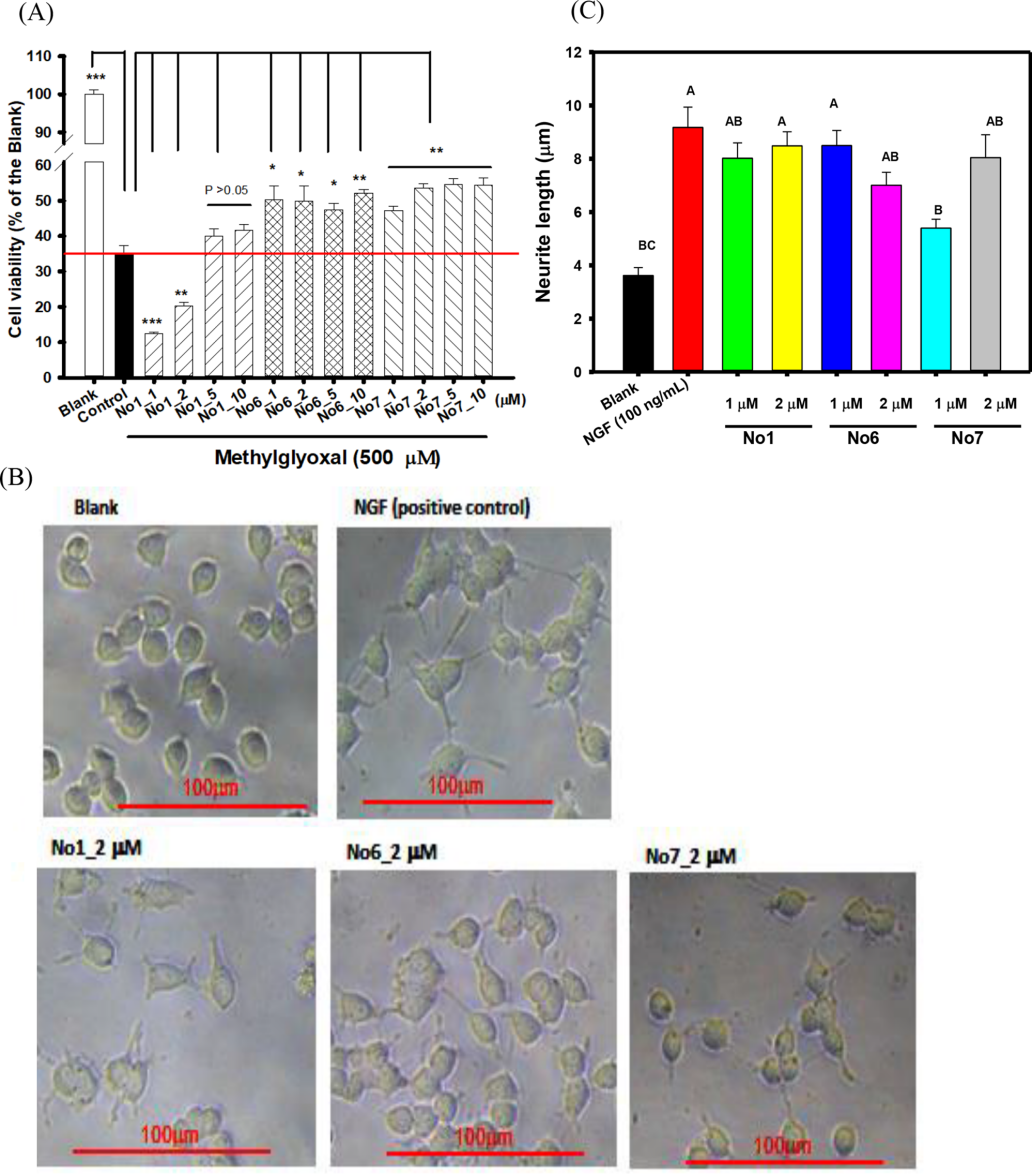
Effects of hispolon (No.1) and its two analogs (No.6 and No.7) treatments (1 to 10 µM) on cell viabilities in methylglyoxal (500 µM)-treated SH-SY5Y neuronal cell models (**A**). The photographs (**B**) and the quantifications of neurite lengths (**C**) of hispolon (No.1) and its two analogs (No.6 and No.7) treatments on neurite outgrowths of PC12 cells, and the nerve growth factor (NGF, 100 ng/mL) was used as the positive control. Values were presented as mean ± SD. The one-way analysis of variance (ANOVA) and the post hoc Tukey’s test were used to compare multiple groups of neurite outgrowths. It was considered statistically a significant difference (*P* < 0.05) when the different uppercase letters in each bar

The supplementary Figure S4(A) showed the cell viabilities of methylglyoxal-treated SH-SY5Y cells at concentrations of 100, 200, 400, 500, and 600 µM by MTT methods. It was clear that the methylglyoxal concentrations higher than 400 µM showed potent cytotoxicities toward SH-SY5Y cells, and the cell viabilities of SH-SY5Y after 400, 500, and 600 µM methylglyoxal treatments were 63%, 39.4%, and 36.6%, respectively. Therefore, the 500 µM methylglyoxal was selected for further experiments. The Fig. 3(A) showed the neuroprotective activities of hispolon (No.1) and its analogs No.6 and No.7 (1, 2, 5, and 10 µM) against 500 µM methylglyoxal-induced SH-SY5Y cell deaths. It was found that the treatment of methylglyoxal at 500 µM could reduce dramatically cell viability from 100% (the blank) to about 38% (the control). The analogs No.6 and No.7 at concentrations of 1, 2, 5, and 10 µM showed to elevate cell viabilities of methylglyoxal-treated SH-SY5Y cells and had significant differences compared to those in the control (*P < 0.05, **P < 0.01), which meant the neuroprotective activities of analogs 6 and 7. The hispolon (No.1) at concentrations of 5 and 10 µM showed to elevate cell viabilities of methylglyoxal-treated SH-SY5Y cells, however, no significant difference was found compared to those in the control (*P* > 0.05). The supplementary Figure S4 (B) showed the photographs of morphologies of methylglyoxal-treated SH-SY5Y cells with or without hispolon (No.1), analog No.6, and analog No.7 treatments at concentration of 10 µM. It was clear that the morphologies of the differentiated SH-SY5Y cells of pyramidal shaped cells with several neurites were lost after methylglyoxal treatments as the same previously reported (Kovalevich and Langford 2013). The treatments of analog No.6, and analog No.7 showed to recover parts of methylglyoxal-damaged neuritic lengths and restore pyramidal shapes (arrow indicated).

The PC12 cell lines (the adrenal gland pheochromocytoma) were used as models for neurite outgrowths, and NGF was used as the positive control to cease proliferation and transform to generate apparent neurites with neuronal markers (Liao et al. 2012). Figure 3(B) showed the photographs of hispolon (No.1), analog No.6, or analog No.7 treatments alone on stimulations of neurite outgrowths of PC12 cells in vitro. It was clear that the NGF could induce PC12 cell transformations to form neuronal phenotypes with apparent neurites after 3-day treatments. The treatments of hispolon (No.1), analog No.6, or analog No.7 also showed the similar NGF effects to transform and stimulate neurite outgrowths of differentiated PC12 cells and had apparent neurites. Figure 3(C) showed the quantification of neurite lengths (µm) of PC12 cells after 3-day treatments by the image J software, which was the general method following the previous report (Liao et al. 2012). The un-differentiated PC12 cells (the blank) exhibited very short neurites with average neurite length of 3.62 ± 1.62 μm, and the NGF-induced differentiations of PC12 cells with average neurite lengths of 9.18 ± 4.87 μm. The hispolon (No.1) treatments at 1 and 2 µM induced differentiations of the PC12 cells with average neurite lengths of 8.02 ± 3.14 and 8.49 ± 3.61 μm, respectively. The analog No.6 treatments at 1 and 2 µM induced differentiations of the PC12 cells with average neurite lengths of 8.50 ± 3.48 and 7.01 ± 3.11 μm, respectively. The analog No.7 treatments at 1 and 2 µM induced differentiations of the PC12 cells with average neurite lengths of 5.40 ± 1.96 and 8.05 ± 4.69 μm, respectively. Based on the analyses of one-way ANOVA and Tukey’s tests, the hispolon (No.1) at 1 and 2 µM, analog No.6 at 1 and 2 µM, and analog No.7 at 2 µM showed to enhance neurite lengths of differentiated PC12 cells and had similar stimulated activities to those of NGF at 100 ng/ml, which showed significant differences compared to those in the blank (*P* < 0.05).

### Effects of hispolon and its two analogs on improving cognitive dysfunctions in scopolamine-induced ICR mice

**Fig. 4:**
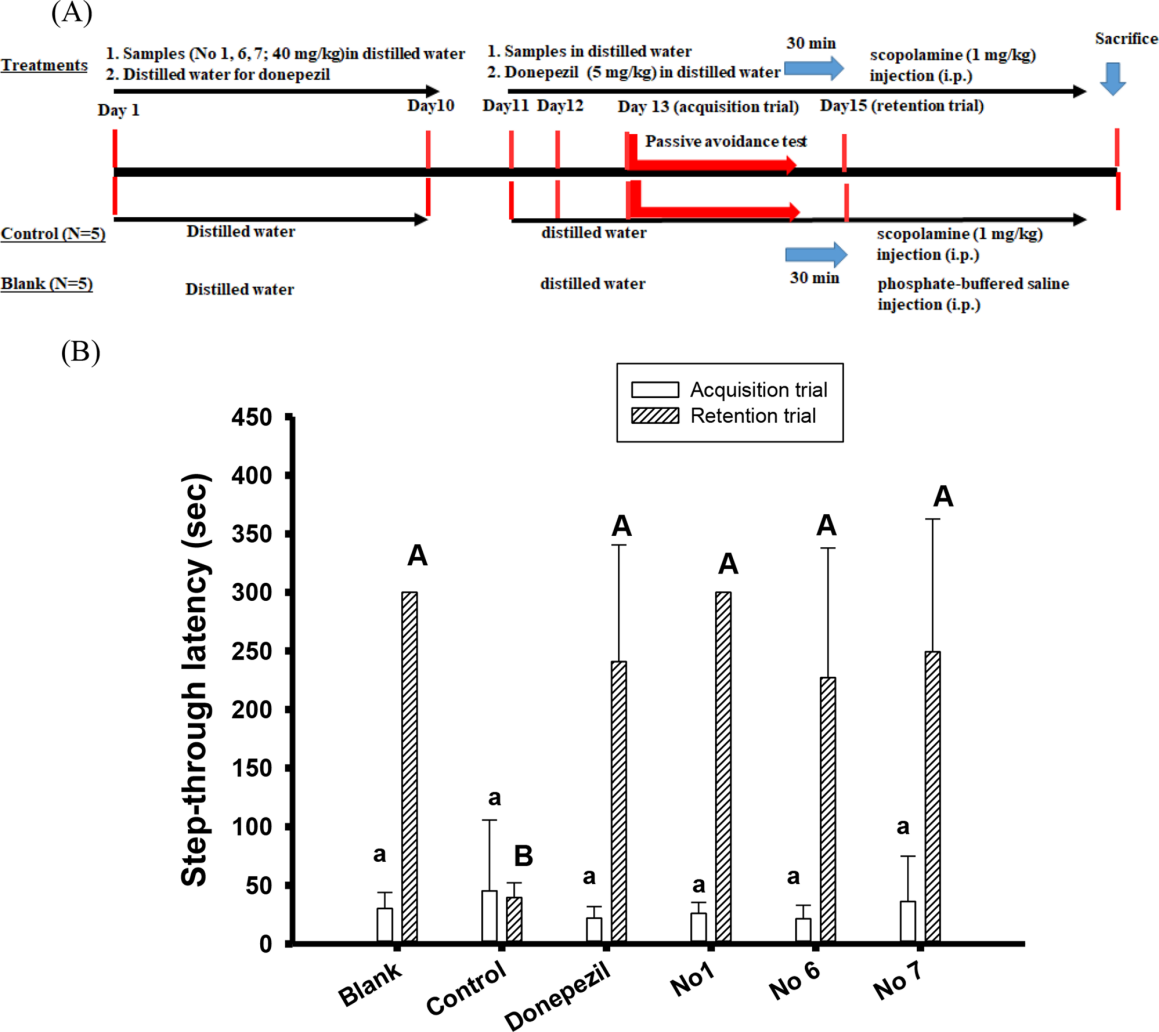
The experimental protocol (**A**) and the step-through latency (sec) of acquisition trial and retention trial in passive avoidance test in the scopolamine-induced ICR mice (**B**). The daily pre-oral administrations of hispolon (No.1) and its two analogs (No.6 and No.7) at concentration of 40 mg/kg, and the donepezil (5 mg per kg of body weight) was used as the positive control. The results of animal experiments were expressed as mean ± SD. The one-way analysis of variance (ANOVA) and the *post hoc* Tukey’s test were used to compare multiple groups of step-through latency (sec) in scopolamine-induced ICR mice. It was considered statistically a significant difference (*P* < 0.05) when the different uppercase letters (for the retention trial) or lowercase (for the acquisition trial) in each bar

The cognitive dysfunctions of ICR mice induced by scopolamine were used as animal models, and the improved learning and memory functions were evaluated by passive avoidance tests. The protocol for the animal experiment was shown in the Fig. 4(A), and the pre-oral administrations of hispolon (No.1), analog No.6, or analog No.7 (40 mg/kg) once a day for 10 days, and then cognitive dysfunction inductions together with sample administrations were used as the preventive modes. Figure 4(B) showed the step-through latency (the staying time in the light box, sec) of the passive avoidance tests at day 13 (the acquisition trial) and day 15 (the retention trial). Each mouse in the different groups at the acquisition trial ran quickly into the dark environment and showed no significant difference (*P* > 0.05) among groups in the step-through latency. After being received the electric feet shocks at day 13 and day 14, the step-through latency of the mouse in each group at day 15 (the retention trial) was recorded. It was clear that mice in the control group also showed to run quickly into the dark environment which the electric foot shocks showed no effects on the learning and memory behaviours of mice in the control. However, mice in the blank, donepezil group, or pre-treated groups of hispolon (No.1), analog No.6 and analog No.7 showed to prolong the staying time in the light box (step-through latency) after being received the electric foot shocks, and showed significant differences (*P* < 0.05) compared to those in the control. It was noted that mice received 10-day pre-treated hispolon (No.1), analog No.6 or analog No.7 showed similar learning behaviours and had no significant difference (*P* > 0.05) in the retention trial to those in the positive control of donepezil.

## Discussion

The present results showed the first time that hispolon, one of bioactive phenolic compounds from a fungus sang-huang (*Phellinus linteus*), and its two analogs exhibited proof-of-concept from in vitro to in vivo impacts to ameliorate cognitive dysfunctions in scopolamine-induced ICR mice and also showed neuroprotective activities and neurite outgrowth stimulated activities. It was reported that the dual activities of Aβ clearance and reactive oxygen species (ROS) scavenging nanoparticles exhibited improved cognitive functions in APP/PS1 transgenic mice, which might be potential therapeutic strategy in AD treatments (Zhang et al. 2024). It was reported that the oxidative stress in brain cells induced by ROS might trigger mitochondrial dysfunction, and ROS-induced Aβ peptide aggregations and microglia activation linked to impaired cognition in AD and ROS-mediated aging in the brain (Manoharan et al. 2016). In the present results, the multifaceted targets of hispolon and its analogs in free radical scavenging activities, anti-nitric oxide productions, inhibitions against AChE and Aβ_1 − 42_ aggregations might be new routes for AD treatments in developing daily ingestions of functional foods or as lead compounds for medical therapies.

The ORAC assay was mainly based on the hydrogen atom transfer (HAT) mechanism, and DPPH radical scavenging assay was relied on a single electron transfer (SET) mechanism, and HAT and SET mechanisms also be performed in parallel in assay systems (Huang et al. 2005; Prior et al. 2005). In the present results, the ORAC activities of hispolon (No.1) and its six analogs at 2.5 µM could be classified into two groups based on IC_50_, one was about 50 µM equivalents of Trolox of the analogs of No.1, No.3, No.4, and No.7; the other was about 30 µM equivalents of Trolox of the analogs of No.2, No.5, and No.6 (Fig. 1C). The DPPH radical scavenging activities of hispolon (No.1) and its six analogs could be classified into two groups based on IC_50_ (Fig. 1B), one was the analogs of No.1, No.2, No.3, No.6, and No.7, which the hydroxy groups in phenyl ring were two to three; the other was the analogs of No.4 and No.5, which the hydroxy groups in phenyl ring were one or zero. It was clear that the numbers of hydroxy groups in the phenyl ring could influence the DPPH radical scavenging activities. The hispolon contained keto-form and enol-form structures. The main structural differences among hispolon and its analogs were numbers and positions of hydroxy and methoxy groups in the phenyl ring. The redox transformations of diphenols were occurred mainly in the *ortho-* and *para-* but not *meta-*configuration of the hydroxy groups (Zenkov et al. 2013). Therefore, it was suggested that three hydroxy groups (No.2 and No.6) or three methoxy groups (No.5) in the phenyl ring had reduced ORAC activities, and no redox transformation of the three methoxy groups (No.5) in the phenyl ring also showed the lowest DPPH scavenging activities. Previously, under no cytotoxic concentrations, the hispolon (5, 10, and 20 µM) showed anti-inflammatory and anti-apoptotic activities by inhibiting nitric oxide productions and iNOS protein expressions and elevating heme oxygenase-1 protein expressions in lipopolysaccharide (10 ng/ml)-treated BV-2 microglia cells (Wu et al. 2017). The orders of anti-inflammatory activities of NF-κB activation of curcumin-related and hispolon-related synthetic compounds were bisdemethylcurcumin (also named as tetramethoxycurcumin) ≅ hispolon (No.1) > analog No.2 > curcumin > analog No.4 > analog No.3 (Ravindran et al. 2010). Though hispolon (5, 10, and 20 µM) showed cytotoxicities toward RAW264.7 macrophages, the additions of hispolon lowered than 2.5 µM and its six analogs at concentrations of 2.5 to 20 µM showed dose-dependent anti-nitric oxide productions in the present results (Supplementary Figure S2), together with the anti-inflammatory and anti-apoptotic activities of hispolon (5, 10, and 20 µM) in LPS-treated BV-2 microglia cells (Wu et al. 2017), made possibilities to reduce neuro-inflammations by using hispolon and its analogs in AD progressions.

The main targets against the cognitive declines in AD pathology were AChE in cholinergic hypothesis (McGleenon et al. 1999; Craig et al. 2011; Čolović et al. 2013) and the different forms of Aβ_1 − 42_ peptides aggregations in amyloid cascade hypothesis (Querfurth and LaFerla 2010; Selkoe and Hardy 2016). The present results showed that the hispolon (No.1) and its analogs, except No.5 (three methoxy groups in the phenyl ring) and No.4 (two methoxy groups in C-3 and C-5 of the phenyl ring), showed dual-targeted activities against AChE activities and Aβ_1 − 42_ peptide aggregations in vitro. The in silico molecular dockings revealed the binding interactions of hispolon (No.1) and analogs of No.6 and No.7 with AChE and the core segments of the Aβ_1 − 42_ peptide (Fig. 2). Previously, the donepezil, the AChE-targeted drug, was used as a standard compound to analyze the interactions among key AA residues of AChE, which the docking pose showed that the donepezil/AChE complex included the hydrogen bonds with AA residues of F295 and S293; the π–π stacked interactions with AA residues of W286, Y341, and W86; the π-alkyl interactions with AA residues Y337 and F338, which these interactions stabilized the donepezil/AChE complex and showed the potent inhibitory activities (Liu et al. 2020; Sie et al. 2023a, b). The AChE comprised two substrate-binding sites, one was catalytic anionic sites (CAS) for the catalysis of ACh hydrolysis; the other was peripheral anionic site (PAS) for allosteric regulations via binding ACh to hinder choline exits from CAS (Colletier et al. 2006). It was found that hispolon (No.1), analog No.6 and analog No.7 were posed in silico into CAS regions of AChE and formed interactions with key amino acids, which resulted in AChE inhibitions (Fig. 2A and C). The stabilities of complex of analog No.6/AChE exhibited the lowest interaction energy (-38.5581 Kcal/mol) among these three docking models, which also showed the highest AChE inhibitory activities among test samples in the present study. The interaction energy of analog No.6/AChE complex (-38.5581 Kcal/mol) showed higher than that of docking pose of scirpusin B/AChE complex (-39.6931 Kcal/mol) and lowered than that of docking pose of piceatannol/AChE complex (-36.0156 Kcal/mol), which the stilbene derivatives of scirpusin B and piceatannol exhibited dual-targeted activities of anti-AChE and Aβ aggregations (Sie et al. 2023b). It was noted that with the structural skeletons similar to that of half-curcumin or demethylcurcumin (Liu et al. 2020), hispolon and its analogs occupied mainly at the CAS region of AChE, which resulted in the loss of the π–π stacked interactions with AA residue W286 and hydrogen bonds with AA residue S293 in the PAS region of AChE. It was reported that the AA residues 16 to 21 of the Aβ_1 − 42_ peptide were the core segments of Aβ self-aggregations (Enache et al. 2016). In the present study, it was found that hispolon (No.1), analog No.6 and analog No.7 were interacted in silico with AA residues V18 to E22 of Aβ_1 − 42_ (Fig. 2D and F), which resulted in the anti-Aβ_1 − 42_ aggregations. The lowest interaction energy of docking pose of analog No.7/Aβ_1−42_ complex (-20.3564 Kcal/mol) showed to act as potent breakers in Aβ self-aggregations. It was noted that the interaction energy of docking pose of analog No.7/Aβ_1 − 42_ complex were higher than those of scirpusin B/Aβ_1−42_ complex (-35.6229 Kcal/mol) and piceatannol/Aβ_1−42_ complex (-25.1388 Kcal/mol) (Sie et al. 2023b).

The present results showed that methylglyoxal at 500 µM showed a significant cytotoxicity toward SH-SY5Y neuronal cell models, and analogs of No.6 and No.7 showed to elevate significantly cell viabilities (Fig. 3A). The reduced cell viabilities of SH-SY5Y cells at low concentrations of hispolon (No.1) co-treated with methylglyoxal might be from a pro-oxidant of hispolon (No.1) to induce ROS during cell cultures (Ravindran et al. 2010). The hispolon and its two analogs of No.6 and No.7 had stimulated effects on neurite outgrowths of differentiated PC12 cells as similar to those of NGF activities (Fig. 3B and C). The major constituents of curcuminoids of curcumin, demethoxycurcumin, and bisdemethoxycurcumin, showed to promote neurite outgrowth in PC12 cells via activating p-ERK1/2 and p-CREB in MAPK/ERK-dependent and PKC-dependent pathways to enhance growth-associated protein-43 (GAP-43) protein and mRNA overexpressions (Liao et al. 2012). The NGF was reported to be the key modulators of neurite outgrowths during nervous developments, the insufficiency of NGF might associate with AD, and the flavonol aglycone of isorhamnetin showed similar NGF activities to promote PC12 neurite outgrowths by protein over-expressions of different molecular weights of neurofilaments (NFs), such as NF68, NF160, and NF200 (Xu et al. 2012). The promoting neurite outgrowths of hispolon (No.1) and its two analogs of No.6 and No.7 in the present PC12 cell models might also upregulate over-expressions of GAP-43 and NFs in MAPK/ERK-dependent pathways, which will be investigated in the future.

The scopolamine-induced mice with impaired cognition were frequently used in AD animal models and clinical trials for investigating efficacy of AChE inhibitors, which scopolamine occupied the muscarinic receptors as an acetylcholine antagonist (McGleenon et al. 1999; Craig et al. 2011; Weinreb et al. 2016). It was found that scopolamine-injected animal models could increase AChE activities, ROS and inflammatory levels in the brain (Kaur et al. 2015). Therefore, the proof-of-concept in vivo scopolamine-induced ICR mice showed impacts of hispolon (No.1) and its analogs No.6 and No.7 in ameliorating cognitive dysfunctions in AD evaluated by the passive avoidance test. The bioavailabilities and pharmacokinetic properties of hispolon and its analogs in animal models were rare, and the hispolon-loaded liquid crystalline nanoparticles delivered drug, showed almost five-folds bioavailabilities (area under curve) in pharmacokinetic studies of Wistar rats compared to those of the hispolon suspensions, and the main distribution were found in the liver and spleen (Ansari et al. 2022). It was also reported that the delivered systems of nanoformulated curcumin could penetrate blood-brain barrier to target brain tissues (Sun et al. 2010; Tsai et al. 2011), which might apply to hispolon or its analogs with structures similar to that of half-curcumin to increase bioavailability to delay cognition declines in AD treatments.

## Conclusions

In this study, multifaceted targets of hispolon and its analogs No.6 and No.7 in free radical scavenging activities, anti-nitric oxide productions, inhibitions against AChE and Aβ_1 − 42_ aggregations, neurite outgrowth stimulated activities, and learning behavior improvements in scopolamine-induced cognitive impairment mouse models might be new routes for AD treatments. The fungus sang-huang is a well-known medicinal mushroom for centuries, which hispolon is one of active phenolic compounds with several functions. It will be beneficial to develop hispolon-enrich sang-huang extracts as functional foods for daily uses or the active component of hispolon as a lead compound to develop derivatives for medical therapies.

## Electronic supplementary material

Below is the link to the electronic supplementary material.

Supplementary Material 1

## Data Availability

All data has been provided with the manuscript. If any additional information is required, then the corresponding author can be contacted.

## References

[CR1] Allaman I, Bélanger M, Magistretti PJ (2015) Methylglyoxal, the dark side of glycolysis. Front Neurosci 9:23. 10.3389/fnins.2015.00023 25709564 10.3389/fnins.2015.00023 PMC4321437

[CR2] Alzheimer’s & Association (2024) Medications for memory, cognition and dementia-related behaviors. https://www.alz.org/alzheimers-dementia/treatments/medications-for-memory

[CR3] Alzheimer’s Association Report (2023) 2023 Alzheimer’s disease facts and figures. Alzheimer’s Dement 19(4):1598–1695. 10.1002/alz.13016 36918389 10.1002/alz.13016

[CR4] Ansari MJ, Rahman M, Alharbi KS, Altowayan WM, Ali AMA, Almalki WH, Barkat MA, Singh T, Nasar S, Akhter MH, Beg S, Choudhry H (2022) Hispolon-loaded liquid crystalline nanoparticles: development, stability, in vitro delivery profile, and assessment of hepatoprotective activity in hepatocellular carcinoma. ACS Omega 7(11):9452–9464. 10.1021/acsomega.1c06796 35350323 10.1021/acsomega.1c06796 PMC8945187

[CR5] Arndt JW, Qian F, Smith BA, Quan C, Kilambi KP, Bush MW, Walz T, Pepinsky RB, Bussière T, Hamann S, Cameron TO, Weinreb PH (2018) Structural and kinetic basis for the selectivity of aducanumab for aggregated forms of amyloid-β. Sci Rep 8:6412. 10.1038/s41598-018-24501-0 29686315 10.1038/s41598-018-24501-0 PMC5913127

[CR6] Chang HY, Sheu MJ, Yang CH, Lu TC, Chang YS, Peng WH, Huang SS, Huang GJ (2011) Analgesic effects and the mechanisms of anti-inflammation of hispolon in mice. Evid Based Complement Alternat Med 2011:478246. 10.1093/ecam/nep027 19349477 10.1093/ecam/nep027 PMC3136186

[CR7] Chen H, Tian T, Miao H, Zhao YY (2016) Traditional uses, fermentation, phytochemistry and pharmacology of *Phellinus linteus*: a review. Fitoterapia 113:6–26. 10.1016/j.fitote.2016.06.009 27343366 10.1016/j.fitote.2016.06.009

[CR8] Chen LG, Lin SY, Lee YS, Wang CC, Hou WC (2021) Hydrolysable tannins exhibit acetylcholinesterase inhibitory and anti-glycation activities in vitro and learning and memory function improvements in scopolamine-induced amnesiac mice. Biomedicines 9(8):1066. 10.3390/biomedicines9081066 34440270 10.3390/biomedicines9081066 PMC8394356

[CR9] Chen LG, Wang CC, Lee YS, Sie YY, Chang CI, Hou WC (2022) Vitisin A, a resveratrol tetramer, improves scopolamine-induced impaired learning and memory functions in amnesiac ICR mice. Biomedicines 10(2):273. 10.3390/biomedicines10020273 35203483 10.3390/biomedicines10020273 PMC8869728

[CR10] Colletier JP, Fournier D, Greenblatt HM, Stojan J, Sussman JL, Zaccai G, Silman I, Weik M (2006) Structural insights into substrate traffic and inhibition in acetylcholinesterase. EMBO J 25(12):2746–2756. 10.1038/sj.emboj.7601175 16763558 10.1038/sj.emboj.7601175 PMC1500847

[CR11] Čolović MB, Krstić DZ, Lazarević-Pašti TD, Bondžić AM, Vasić VM (2013) Acetylcholinesterase inhibitors: pharmacology and toxicology. Curr Neuropharmacol 11(3):315–335. 10.2174/1570159X11311030006 24179466 10.2174/1570159X11311030006 PMC3648782

[CR12] Craig LA, Hong NS, McDonald RJ (2011) Revisiting the cholinergic hypothesis in the development of Alzheimer’s disease. Neurosci Biobehav Rev 35(6):1397–1409. 10.1016/j.neubiorev.2011.03.001 21392524 10.1016/j.neubiorev.2011.03.001

[CR13] Cummings J, Osse AML, Cammann D, Powell J, Chen J (2024) Anti-amyloid monoclonal antibodies for the treatment of Alzheimer’s disease. BioDrugs 38(1):5–22. 10.1007/s40259-023-00633-2 37955845 10.1007/s40259-023-00633-2 PMC10789674

[CR14] Enache TA, Chiorcea-Paquim AM, Oliveira-Brett AM (2016) Amyloid-β peptides time-dependent structural modifications: AFM and voltammetric characterization. Anal Chim Acta 926:36–47. 10.1016/j.aca.2016.04.015 27216391 10.1016/j.aca.2016.04.015

[CR15] Fortea J, Pegueroles J, Alcolea D, Belbin O, Dols-Icardo O, Vaqué-Alcázar L, Videla L, Gispert JD, Suárez-Calvet M, Johnson SC, Sperling R, Bejanin A, Lleó A, Montal V (2024) APOE4 homozygozity represents a distinct genetic form of Alzheimer’s disease. Nat Med 30:1284–1291. 10.1038/s41591-024-02931-w 38710950 10.1038/s41591-024-02931-w PMC13310155

[CR16] Gella A, Durany N (2009) Oxidative stress in Alzheimer disease. Cell Adh Migr 3(1):88–93. 10.4161/cam.3.1.7402 19372765 10.4161/cam.3.1.7402 PMC2675154

[CR17] Hsieh PW, Wu JB, Wu YC (2013) Chemistry and biology of *Phellinus Linteus*. BioMedicine 3(3):106–113. 10.1016/j.biomed.2013.01.002

[CR18] Hu R, Cao Q, Sun Z, Chen J, Zheng Q, Xiao F (2018) A novel method of neural differentiation of PC12 cells by using Opti-MEM as a basic induction medium. Int J Mol Med 41(1):195–201. 10.3892/ijmm.2017.3195 29115371 10.3892/ijmm.2017.3195 PMC5746309

[CR19] Huang D, Ou B, Prior RL (2005) The Chemistry behind antioxidant capacity assays. J Agric Food Chem 53(6):1841–1856. 10.1021/jf030723c 15769103 10.1021/jf030723c

[CR20] Huang GJ, Deng JS, Chiu CS, Liao JC, Hsieh WT, Sheu MJ, Wu CH (2012) Hispolon protects against acute liver damage in the rat by inhibiting lipid peroxidation, proinflammatory cytokine, and oxidative stress and downregulating the expressions of iNOS, COX-2, and MMP-9. Evid Based Complement Alternat Med 2012:480714. 10.1155/2012/480714 22013489 10.1155/2012/480714 PMC3195309

[CR21] Jack CR Jr, Lowe VJ, Weigand SD, Wiste HJ, Senjem ML, Knopman DS, Shiung MM, Gunter JL, Boeve BF, Kemp BJ, Weiner M, Petersen RC, the Alzheimer’s Disease Neuroimaging Initiative (2009) Serial PIB and MRI in normal, mild cognitive impairment and Alzheimer’s disease: implications for sequence of pathological events in Alzheimer’s disease. Brain 132(5):1355–1365. 10.1093/brain/awp062 19339253 10.1093/brain/awp062 PMC2677798

[CR22] Kaur R, Mehan S, Khanna D, Kalra S (2015) Ameliorative treatment with ellagic acid in scopolamine induced Alzheimer’s type memory and cognitive dysfunctions in rats. Austin J Clin Neurol 2(6):1053

[CR23] Kikuchi S, Shinpo K, Takeuchi M, Yamagishi S, Makita Z, Sasaki N, Tashiro K (2003) Glycation - a sweet tempter for neuronal death. Brain Res Rev 41(2–3):306–323. 10.1016/S0165-0173(02)00273-4 12663085 10.1016/s0165-0173(02)00273-4

[CR24] Kim DH, Yang BK, Jeong SC, Park JB, Cho SP, Das S, Yun JW, Song CH (2001) Production of a hypoglycemic, extracellular polysaccharide from the submerged culture of the mushroom, *Phellinus linteus*. Biotechnol Lett 23:513–517. 10.1023/A:1010312513878

[CR25] Kovalevich J, Langford D (2013) Considerations for the use of SH-SY5Y Neuroblastoma cells in Neurobiology. In: Amini S, White M (eds) Neuronal cell culture. Methods in Molecular Biology, vol 1078. Humana, Totowa, NJ, pp 9–21. 10.1007/978-1-62703-640-5_2 10.1007/978-1-62703-640-5_2 PMC5127451 23975817

[CR26] Kozarski M, Klaus A, Niksic M, Jakovljevic D, Heisper JPFG, Van Griensven LJLD (2011) Antioxidative and immunomodulating activities of polysaccharide extracts of the medicinal mushrooms *Agaricus Bisporus*, *Agaricus Brasiliensis*, *Ganoderma lucidum* and *phellinus linteus*. Food Chem 129(4):1667–1675. 10.1016/j.foodchem.2011.06.029

[CR27] Liao KK, Wu MJ, Chen PY, Huang SW, Chiu SJ, Ho CT, Yen JH (2012) Curcuminoids promote neurite outgrowth in PC12 cells through MAPK/ERK- and PKC-dependent pathways. J Agric Food Chem 60(1):433–443. 10.1021/jf203290r 22145830 10.1021/jf203290r

[CR28] Liu YH, Lee TL, Han CH, Lee YS, Hou WC (2019) Anti–glycation, anti–hemolysis, and ORAC activities of demethylcurcumin and tetrahydroxycurcumin in vitro and reductions of oxidative stress in D–galactose–induced BALB/c mice in vivo. Bot Stud 60:9. 10.1186/s40529-019-0258-x 31250143 10.1186/s40529-019-0258-x PMC6597665

[CR29] Liu YH, Lee CJ, Chen LC, Lee TL, Hsieh YY, Han CH, Yang CH, Huang WJ, Hou WC (2020) Acetylcholinesterase inhibitory activity and neuroprotection in vitro, molecular docking, and improved learning and memory functions of demethylcurcumin in scopolamine-induced amnesia ICR mice. Food Funct 11(3):2328–2338. 10.1039/C9FO02339A 32118214 10.1039/c9fo02339a

[CR30] Lu YL, Liu YH, Chyuan JH, Cheng KT, Liang WL, Hou WC (2012) Antioxidant activities of different wild bitter gourd (*Momordica charantia* L. var. *abbreviata* Seringe) cultivars. Bot Stud 53(2):207–214. https://ejournal.sinica.edu.tw/bbas/content/2012/2/Bot532-04/Bot532-04.html

[CR31] Manoharan S, Guillemin GJ, Abiramasundari RS, Essa MM, Akbar M, Akbar MD (2016) The role of reactive oxygen species in the pathogenesis of Alzheimer’s disease, Parkinson’s disease, and Huntington’s disease: a mini review. Oxid Med Cell Longev 2016(1):8590578. 10.1155/2016/8590578 28116038 10.1155/2016/8590578 PMC5223034

[CR32] McGleenon BM, Dynan KB, Passmore AP (1999) Acetylcholinesterase inhibitors in Alzheimer’s disease. Brit J Clin Pharmacol 48(4):471–480. 10.1046/j.1365-2125.1999.00026.x 10583015 10.1046/j.1365-2125.1999.00026.x PMC2014378

[CR33] Monteiro AR, Barbosa DJ, Remião F, Silva R (2023) Alzheimer’s disease: insights and new prospects in disease pathophysiology, biomarkers and disease-modifying drugs. Biochem Pharmacol 211:115522. 10.1016/j.bcp.2023.115522 36996971 10.1016/j.bcp.2023.115522

[CR34] National Institute on Aging What causes Alzheimer’s disease? https://www.nia.nih.gov/health/what-causes-alzheimers-disease

[CR35] Osborne OM, Naranjo O, Heckmann BL, Dykxhoorn D, Toborek M (2023) Anti-amyloid: an antibody to cure Alzheimer’s or an attitude. iScience 26(8):107461. 10.1016/j.isci.2023.107461 37588168 10.1016/j.isci.2023.107461 PMC10425904

[CR36] Perneczky R, Jessen F, Grimmer T, Levin J, Flöel A, Peters O, Froelich L (2023) Anti-amyloid antibody therapies in Alzheimer’s disease. Brain 146(3):842–849. 10.1093/brain/awad005 36655336 10.1093/brain/awad005

[CR37] Plassman BL, Lang KM, Fisher GG, Heeringa SG, Weir DR, Ofstedal MB, Burke JR, Hurd MD, Potter GG, Rodgers WL, Steffens DC, Willis RJ, Wallace RB (2007) Prevalence of dementia in the United States: the aging, demographics, and memory study. Neuroepidemiol 29(1–2):125–132. 10.1159/000109998 10.1159/000109998 PMC2705925 17975326

[CR38] Prior RL, Wu X, Schaich K (2005) Standardized methods for the determination of antioxidant capacity and phenolics in foods and dietary supplements. J Agric Food Chem 53(10):4290–4302. 10.1021/jf0502698 15884874 10.1021/jf0502698

[CR39] Querfurth HW, LaFerla FM (2010) Alzheimer’s disease. New Eng J Med 362(4):329–344. 10.1056/NEJMra0909142 20107219 10.1056/NEJMra0909142

[CR40] Ravindran J, Subbaraju GV, Ramani MV, Sung B, Aggarwal BB (2010) Bisdemethylcurcumin and structurally related hispolon analogues of curcumin exhibit enhanced prooxidant, anti-proliferative and anti-inflammatory activities. Biochem Pharmacol 79(11):1658–1666. 10.1016/j.bcp.2010.01.033 20138025 10.1016/j.bcp.2010.01.033 PMC2846970

[CR41] Sarfraz A, Rasul A, Sarfraz I, Shah MA, Hussain G, Shafiq N, Masood M, Adem Ş, Sarker SD, Li X (2020) Hispolon: a natural polyphenol and emerging cancer killer by multiple cellular signaling pathways. Environ Res 190:110017. 10.1016/j.envres.2020.110017 32768475 10.1016/j.envres.2020.110017 PMC7406431

[CR42] Selkoe DJ, Hardy J (2016) The amyloid hypothesis of Alzheimer’s disease at 25 years. EMBO Mol Med 8(6):595–608. 10.15252/emmm.201606210 27025652 10.15252/emmm.201606210 PMC4888851

[CR43] Serrano-Pozo A, Frosch MP, Masliah E, Hyman BT (2011) Neuropathological alterations in Alzheimer disease. Cold Spring Harb Perspect Med 1(1):a006189. 10.1101/cshperspect.a006189 22229116 10.1101/cshperspect.a006189 PMC3234452

[CR44] Sie YY, Chen LC, Li CJ, Yuan YH, Hsiao SH, Lee MH, Wang CC, Hou WC (2023a) Inhibition of acetylcholinesterase and amyloid-β aggregation by piceatannol and analogs: assessing in vitro and in vivo impact on a murine model of scopolamine-induced memory impairment. Antioxidants 12(7):1362. 10.3390/antiox12071362 37507902 10.3390/antiox12071362 PMC10376691

[CR45] Sie YY, Chen LC, Li CW, Wang CC, Li CJ, Liu DZ, Lee MH, Chen LG, Hou WC (2023b) Extracts and scirpusin B from recycled seeds and rinds of passion fruits (*Passiflora edulis* var. Tainung 1) exhibit improved functions in scopolamine-induced impaired-memory ICR mice. Antioxidants 12(12):2058. 10.3390/antiox12122058 38136179 10.3390/antiox12122058 PMC10741041

[CR46] Sun M, Gao Y, Guo C, Cao F, Song Z, Xi Y, Yu A, Li A, Zhai G (2010) Enhancement of transport of curcumin to brain in mice by poly (n-butylcyanoacrylate) nanoparticle. J Nanopart Res 12:3111–3122. 10.1007/s11051-010-9907-4

[CR47] Tarawneh R, Holtzman DM (2012) The clinical problem of symptomatic Alzheimer disease and mild cognitive impairment. Cold Spring Harb Perspect Med 2(5):a006148. 10.1101/cshperspect.a006148 22553492 10.1101/cshperspect.a006148 PMC3331682

[CR48] Tsai YM, Chien CF, Lin LC, Tsai TH (2011) Curcumin and its nano-formulation: the kinetics of tissue distribution and blood-brain barrier penetration. Internat J Pharmaceut 416(1):331–338. 10.1016/j.ijpharm.2011.06.030 10.1016/j.ijpharm.2011.06.030 21729743

[CR49] van Dyck CH, Swanson CJ, Aisen P, Bateman RJ, Chen C, Gee M, Kanekiyo M, Li D, Reyderman L, Cohen S, Froelich L, Katayama S, Sabbagh M, Vellas B, Watson D, Dhadda S, Irizarry M, Kramer LD, Iwatsubo T (2023) Lecanemab in early Alzheimer’s disease. N Engl J Med 388(1):9–21. 10.1056/NEJMoa2212948 36449413 10.1056/NEJMoa2212948

[CR50] Wang CC, Lin SY, Cheng HC, Hou WC (2006) Pro-oxidant and cytotoxic activities of atractylenolide I in human promyeloleukemic HL-60 cells. Food Chem Toxicol 44(8):1308–1315. 10.1016/j.fct.2006.02.008 16624472 10.1016/j.fct.2006.02.008

[CR51] Weinreb O, Amit T, Bar-Am O, Youdim MB (2016) Neuroprotective effects of multifaceted hybrid agents targeting MAO, cholinesterase, iron and beta-amyloid in aging and Alzheimer’s disease. Brit J Pharmacol 173(13):2080–2094. 10.1111/bph.13318 26332830 10.1111/bph.13318 PMC4908201

[CR52] Witt A, Macdonald N, Kirkpatrick P (2004) Memantine hydrochloride. Nat Rev Drug Discover 3:109–110. 10.1038/nrd1311 10.1038/nrd1311 15040575

[CR53] World Health Organization (2003) Dementia. https://www.who.int/news-room/fact-sheets/detail/dementia

[CR54] World Health Organization (2024) The top 10 causes of death. https://www.who.int/news-room/fact-sheets/detail/the-top-10-causes-of-death

[CR55] Wu MS, Chien CC, Cheng KT, Subbaraju GV, Chen YC (2017) Hispolon suppresses LPS- or LTA-induced iNOS/NO production and apoptosis in BV-2 microglial cells. Amer J Chin Med 5(8):1649–1666. 10.1142/S0192415X17500896 10.1142/S0192415X17500896 29121802

[CR56] Xu SL, Choi RCY, Zhu KY, Leung KW, Guo AJY, Bi D, Xu H, Lau DTW, Dong TTX, Tsim KWK (2012) Isorhamnetin, a flavonol aglycone from *Ginkgo biloba* L., induces neuronal differentiation of cultured PC12 cells: potentiating the effect of nerve growth factor. Evid Based Complement Alternat Med 2012:278273. 10.1155/2012/278273 22761636 10.1155/2012/278273 PMC3385709

[CR57] Zenkov NK, Menshchikova EB, Tkachev VO (2013) Keap1/Nrf2/ARE redox-sensitive signaling system as a pharmacological target. Biochem (Moscow) 78(1):19–36. 10.1134/S0006297913010033 10.1134/S0006297913010033 23379556

[CR58] Zhang L, Cao K, Xie J, Liang X, Gong H, Luo Q, Luo H (2024) Aβ42 and ROS dual-targeted multifunctional nanocomposite for combination therapy of Alzheimer’s disease. J Nanobiotechnol 22:278. 10.1186/s12951-024-02543-z 10.1186/s12951-024-02543-z PMC11112798 38783363

