# Supplementary material for: Antioxidant, anti-acetylcholinesterase, and anti-amyloid-β peptide aggregations of hispolon and its analogs in vitro and improved learning and memory functions in scopolamine-induced ICR mice
Source: Bot Stud. 2024 Dec 18;65:38. doi: 10.1186/s40529-024-00443-x (PMC11655744; doi:10.1186/s40529-024-00443-x)
Supplement: Supplementary file 1 — Supplementary Material 1 [file 40529_2024_443_MOESM1_ESM.docx]

Supplementary materials

Antioxidant, anti-acetylcholinesterase, and anti-amyloid-β peptide aggregations of hispolon and its analogs in vitro and improved learning and memory functions in scopolamine-induced ICR mice

Chang-Hang Yang†

Cai-Wei Li

Yi-Yan Sie

Liang-Chieh Chen†

Yu-Hsiang Yuan

Wen-Chi Hou^★^

† These two authors contributed equally to this work

^★^Correspondence

**Table S1.** CDOCKER interaction energy for hispolon (No.1) and analogs of No.6 and No.7 docked in AChE and core fragments of Aβ_1-42_ peptide.


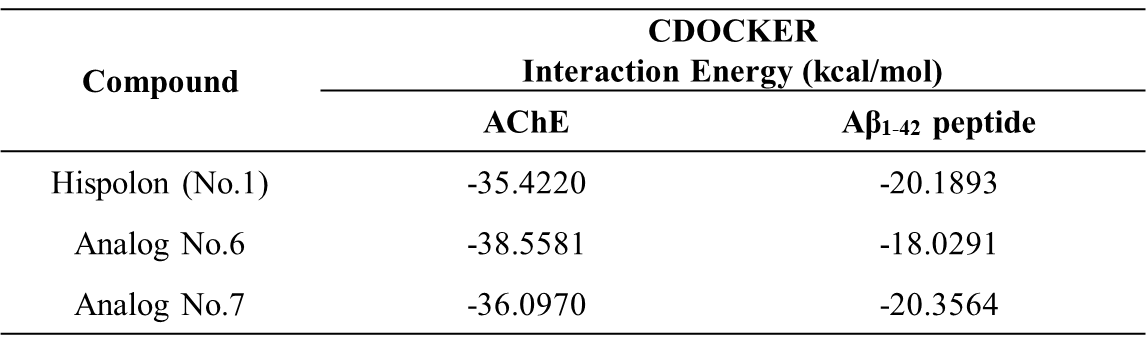


Figure S1

**Figure S1.** The ORAC activities of hispolon (No.1) and its analogs (No.2 to No.7) at concentration of 2.5 μM on inhibiting AAPH-mediated fluorescent decays during 60-min by determining the ratio of Ex480 nm/Em520 nm. The Trolox (5, 10, 20, 40, and 60 μM) were used to plot the standard curve for ORAC assays.

Figure S2


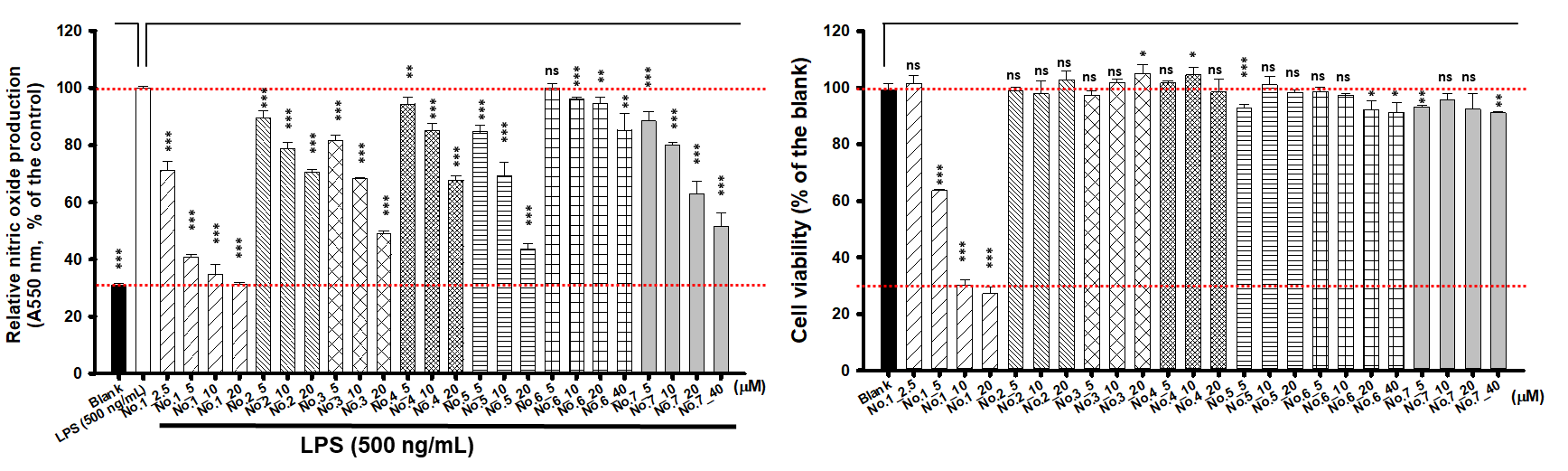


**(A)**

**(B)**

**Figure S2.** The different concentrations of hispolon (No.1) and its analogs (No.2 to No.7) on the (A) inhibition of LPS (500 ng/mL)-induced nitric oxide productions of RAW264.7 macrophages and (B) cell viability (%) of RAW264.7 macrophages. The Student’s *t*-test was used to compare between [(the LPS) vs (sample treatment)] or (the LPS) vs (the Blank)] or between [(the blank) vs (sample treatment)]. It was considered statistically a significant difference when *P* < 0.05 *, or *P* < 0.01**, or *P* < 0.001***; ns, *P* > 0.05.

Figure S3


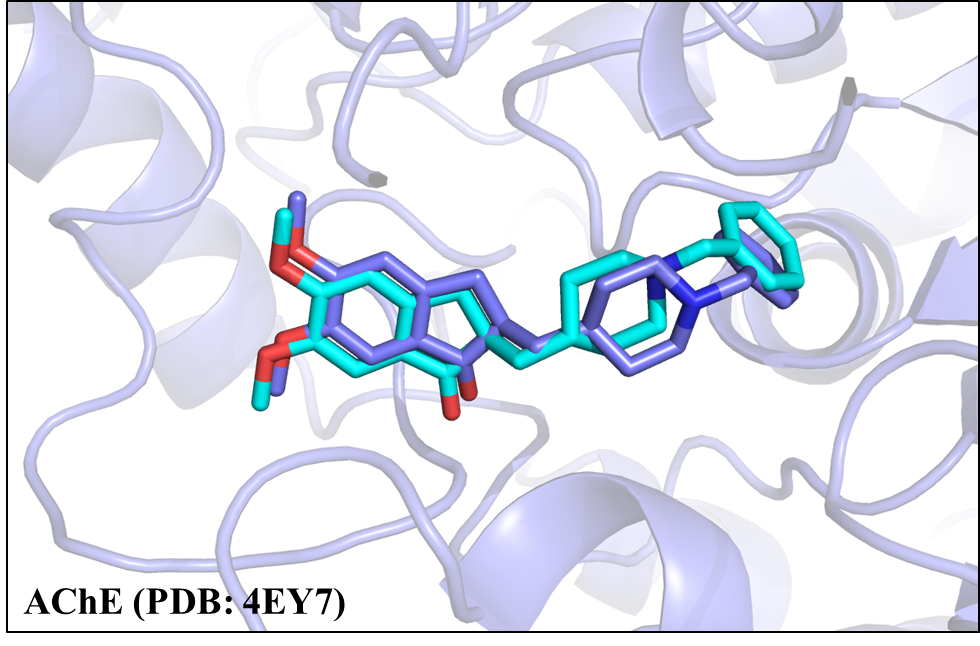


PAS

CAS

**Figure S3**. Validation of the docking protocol by using donepezil. The co-crystallized ligand was re-docked to confirm the docking protocol of AChE. The co-crystallized ligand (light blue) and the docked ligand (cyan) demonstrated strikingly similar poses. The catalytic anionic site (CAS) and the peripheral anionic site (PAS) of AChE.

Figure S4

**(A)**


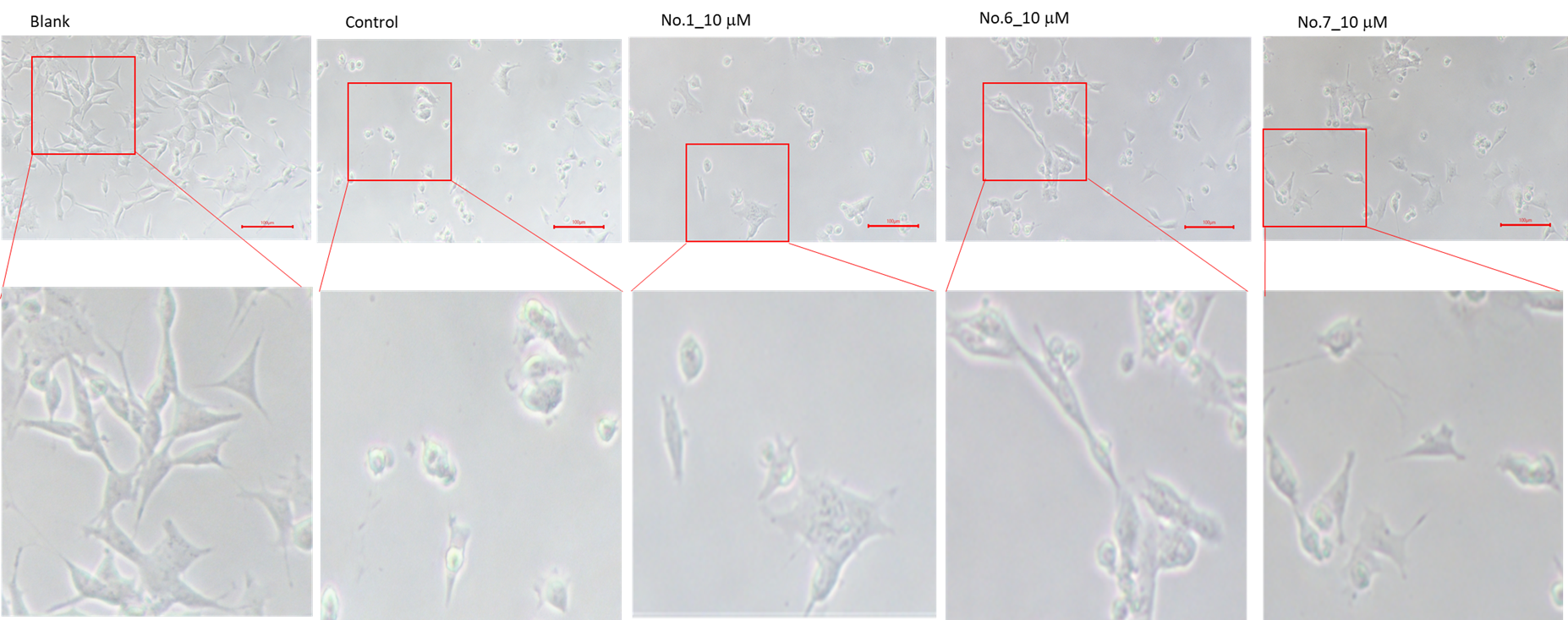


**(B)**

**Figure S4.** (A) Effects of different concentrations of methylglyoxal on SH-SY5Y cell viabilities; ((B) The photographs of hispolon (No.1) and two analogs (No.6 and No.7) treatments on ameliorating 500 μM methylglyoxal-mediated SH-SY5Y cell deaths. The cell morphologies were photographed (200-fold magnifications) and local zooms of images (red square frame) using an inverted microscope (ECLIPSE TS100, Nikon Instruments Inc., Tokyo, Japan). The arrows indicated the recovery of methylgloxal-damaged neurites and restoration of pyramidal shapes.
